# In vitro renal calculi destruction by a high-frequency glow discharge plasma

**DOI:** 10.1038/s41598-022-16702-5

**Published:** 2022-07-25

**Authors:** Sergej V. Belov, Yury K. Danileyko, Roman Y. Pishchalnikov, Sergey V. Gudkov, Alexej V. Egorov, Vladimir I. Lukanin, Vladimir A. Sidorov, Vladimir B. Tsvetkov, Stanislav K. Ali, Sergey V. Kondrashev, Evgeny G. Rotanov, Andrei V. Shakhovskoy, Stepan N. Andreev, Evgeny A. Bezrukov, Petr V. Glybochko

**Affiliations:** 1 Prokhorov General Physics Institute of the Russian Academy of Sciences, Moscow, Russia; 2 Sechenov First Moscow State Medical University, Ministry of Health of the Russian Federation, Moscow, Russia; 3 K. G. Razumovsky Moscow State University of Technologies and Management, Moscow, Russia; 4 Moscow Polytechnic University, Moscow, Russia

**Keywords:** Engineering, Renal calculi, Plasma physics

## Abstract

Despite the progress made in the treatment of nephrolithiasis, the existing methods of renal calculi destruction are not ideal and have both advantages and disadvantages. Considering the process of high-frequency glow discharge formation on the surface of an electrode and in an electrolyte solution, we obtained the results on the destruction of renal calculi in vitro. It was shown that the destruction of kidney stones by glow discharge plasma was caused by several processes—the plasma induced effect of hydrated electrons and shock wave effect of the electrolyte stimulated by electrical breakdowns in the plasma. The plasma generation modes were configured by estimating the thickness of the vapor–gas layer in which the plasma burns. Thus, the average rate of contact destruction of renal calculi was measured depending on the plasma generator input power and time of plasma exposure. We conclude that the method of stone fragmentation by high-frequency electrolyte plasma is rather perspective and can be used in endoscopic urology for percutaneous and transurethral lithotripsy.

## Introduction

Kidney stone disease is also known as nephrolithiasis or ureterolithiasis (stones in the ureters) is one of the most ancient diseases known to medicine^1–4^. The modern methodology for the treatment of stones of the urinary tract consists of their contact destruction or lithotripsy (stone crushing) and lithoextraction (stone evacuation)^5^. For large stones (> 2 cm) and coral kidney stones, percutaneous lithotripsy is used via ultrasound, pneumatic, shock wave or laser exposure^6^. Destruction of calculi by contact, including percutaneous nephrolithotripsy, is the most progressive due to the low invasiveness of the surgical intervention^7^.

The method of lithotripsy is based on the use of various forms of energy impact: mechanical, ultrasonic, laser and others. Accordingly, the implementation of lithotripsy is carried out using ultrasonic, pneumatic, electrokinetic, electrohydraulic, laser and ultrasonic contact lithotriptors or a combination of them. At the same time, each type of lithotripsy has its advantages and disadvantages^8–10^. For example, ultrasound lithotripsy uses only rigid probes and rigid endoscopes, and its scope of application is mainly limited to kidney stones. Contact lithotripsy (pneumatic or electrokinetic lithotripters) is one of the most effective and safe methods of contact destruction of stones^11,12^. At the same time, the use of such lithotripters is also limited by rigid endoscopes, and retrograde stone propulsion in transurethral lithotripsy is a disadvantage of the method^11–13^. Electrohydraulic and laser methods of lithotripsy, being effective methods of contact crushing, can be used with both rigid and flexible endoscopes, which significantly expands the scope of their use in modern urology. However, electrohydraulic lithotripsy causes significantly more complications compared to other methods, since the generated shock wave is capable of damaging the surrounding tissues^11–13^. Laser lithotripsy is less traumatic due to the lack of a ballistic effect; however, it requires more expensive equipment and more time to destroy the stone. At the same time, one of the disadvantages of the method is the presence of a potential danger of laser radiation for others and the possibility of damage to expensive flexible endoscopes due to breakage of the laser fiber^14,15^.

Taking into account the relevance of the lithotripsy procedure in urology, it is of interest to search for new effective and low-cost methods for the destruction of renal calculi. New methods that claim to be promising include plasmakinetic percutaneous lithotripsy based on the destruction of renal calculi using low temperature discharge plasma (LTP). Analysis of modern methods of lithotripsy shows that the method of plasmakinetic destruction of renal calculi is not found in the literature^16^. At the same time, the results of experimental studies show that plasmakinetic destruction of urinary calculi to grains of sand < 500 microns in size can be an effective and low-cost method.

The purpose of this article is: (i) substantiation of the mechanism of plasmakinetic destruction of renal calculi; (ii) discussion of experimental data on the destruction of renal calculi using high-frequency glow discharge; and (iii) evaluation of the effectiveness of the method of plasmakinetic lithotripsy.

## Materials and methods

### Experimental setup

**Figure 1:**
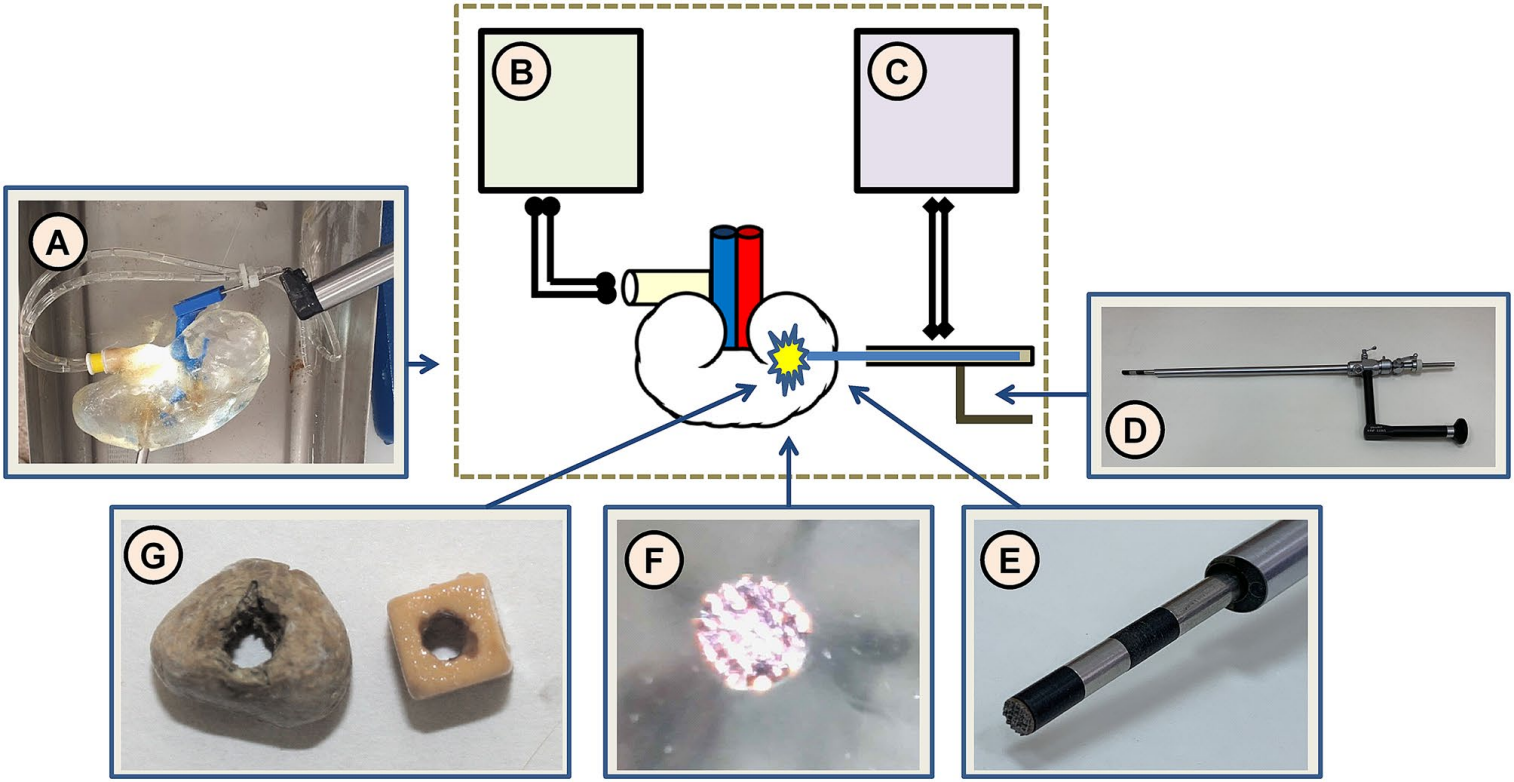
Scheme of the experimental setup: operational field of the experimental setup (**A**), a saline pumper (**B**), a high-frequency current generator (**C**), a urethroscope (**D**); distal part of the urethroscope for plasmakinetic lithotripsy: urethroscope and the plasma electrode (**E**); plasma generation on the distal part of the probe (**F**); the samples of a dummy kidney stone and a natural kidney stone after plasma treatment (**G**).

To assess the plasmakinetic destruction of renal calculi, a study of plasma-chemical etching of renal calculi was carried out. LTP exposure was performed using a developed experimental instrument for plasmakinetic lithotripsy, the essential parts of which are shown in Fig. 1.

In the manufacture of the working part of the instrument for plasmakinetic lithotripsy, stainless steel (18XH9T, Russia) was used for the insulating the gasket (zirconium ceramics) and passive electrode (stainless steel). The active electrode had a diameter of 6 mm with pyramidal protrusions 0.5 mm high, located with a step of 1.0 mm over the entire working surface of the electrode.

The object of the study was samples of natural and dummy urinary stones. As a dummy stone, samples of artificial stone “Begostone” (1200 HU) were used, imitating “soft” stones (urates, phosphates, etc.). In vitro experiments on the destruction of natural renal calculi were carried out on oxalate (hard) and phosphate (soft) stones. Renal calculi based on calcium oxalate monohydrate (1400–1500 HU) were studied as a natural, hard stone. The stone sample was placed in a transparent cuvette filled with saline. A Karl Storz Hopkins II urethroscope was used to place the electrode into a silicon kidney.

The destruction process was recorded by photography. After the specified time of exposure to LTP, the surface of the stone was examined under a microscope, after which the experiment continued. An ESP-01 electrosurgical apparatus (manufactured by ElePS, Kazan, Russia) was used as a high-frequency current generator. The amplitude of the RF voltage supplied to the active electrode was regulated in the range from 100 to 600 V. LeCroy WaveSurfer 64MXs oscilloscope was used to detect the voltage and current of burning plasma. When the working part of the active electrode touched the surface of the sample under study, the LTP plasma ignited on its working part, causing the plasma chemical decomposition of the stone. The treatment was carried out in 0.9% NaCl solution (physiological solution) in a flow-through mode at a temperature of 36ºC.

### High-frequency glow-type discharge on the surface of a metal electrode in an electrolyte solution

**Figure 2:**
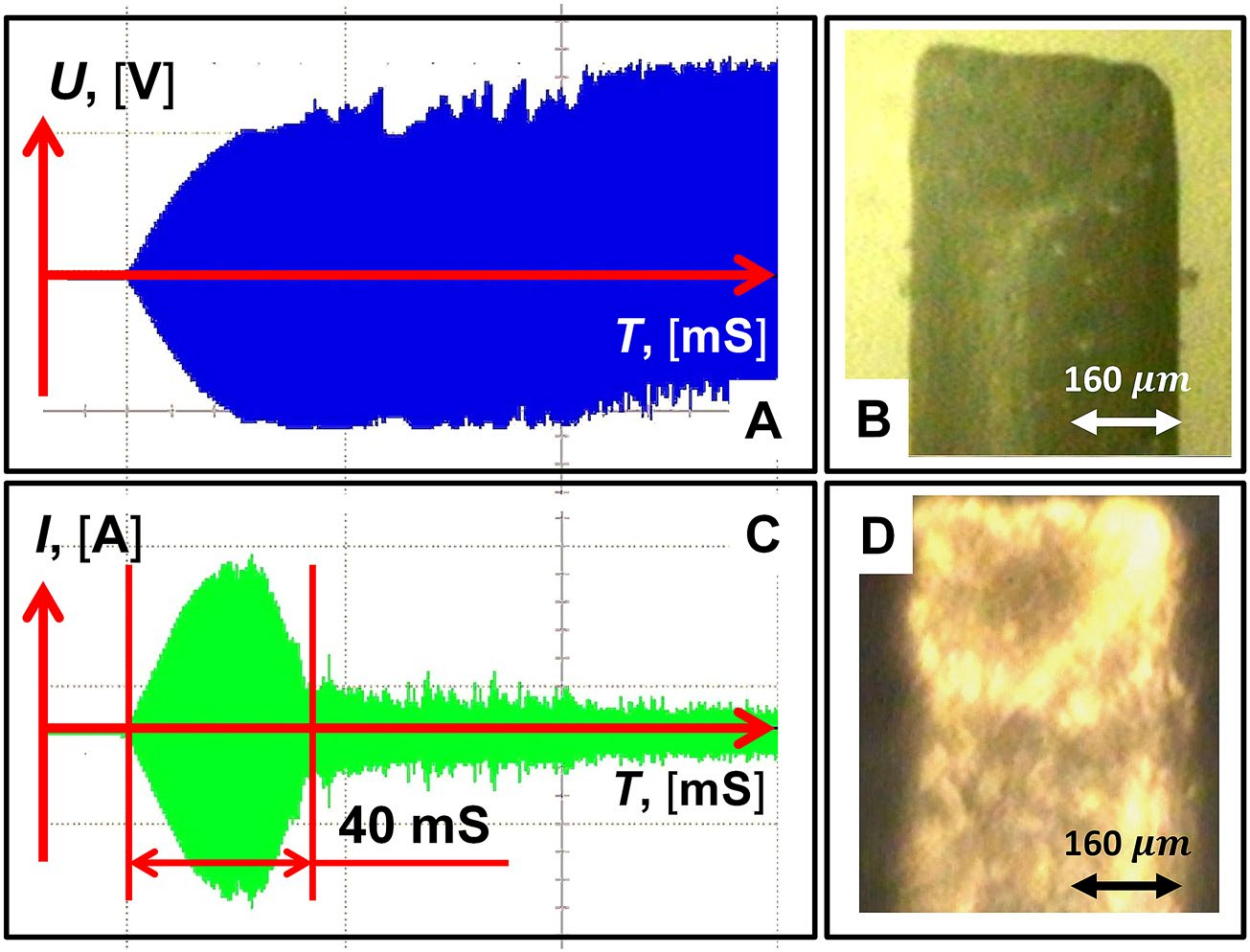
The voltage (**A**) and current (**C**) flowing through the electrode near the plasma discharge ignition threshold are shown. The characteristic time of enveloping the electrode with plasma is approximately 20 ms (half of the current band). Time-integrated image of a tungsten electrode with a diameter of 500 µm over a period of 20 ms: no plasma (**B**) and plasma on the electrode (**D**).

Consider an electrical circuit consisting of two metal electrodes immersed in a solution of a strong electrolyte of low concentration (saline). An electrolyte solution with a specific resistance of ζ = 4.3 Ohm⋅m, together with the electrodes, is placed in a glass laboratory cell. One of the electrodes, made of tungsten and shaped like a cylinder with a diameter of 2R = 500 μm, is immersed in the solution to a depth of L = 10 mm. In what follows, this electrode will be called active, since it is on its surface that plasma processes will take place. The second electrode is passive. It has a surface area many times larger than the area of the active electrode and provides a closed electrical circuit. High-frequency voltage is applied to the electrodes with a frequency of 440 kHz and an amplitude that rises to 500 V over 20 ms. Under the action of the RF current, a plasma discharge appears on the surface of the active electrode, which has a threshold character^17–23^. In this case, the initial gas medium in which the LTP ignites is saturated water vapor formed on the surface of the active electrode with atmospheric pressure and a temperature close to 100 °C. For an experimental assessment of the thickness of the plasma layer, Fig. 2 shows photographs of an electrode (diameter 500 μm) in an electrolyte solution without plasma (panel B) and with plasma on its surface (panel D). Comparison of the images makes it possible to estimate the thickness of the plasma layer, which does not exceed the value (10–30 microns) after its appearance.

Let us qualitatively consider the formation of a vapor layer on the active electrode, which is necessary for the appearance of a glow discharge. As can be seen from Fig. 2D, the plasma discharge is localized in a thin vapor layer on the active electrode, the formation of which can be compared with film boiling^24^. However, in the case under consideration, there is a significant difference in the mechanism of the formation of a thin vapor layer from the case of film boiling on a heated surface. This difference is due to the fact that overheating of the electrolyte to values leading to boiling does not occur due to the thermal head from the hot surface^24^, but due to volumetric heat losses in the electrolyte at the surface of the electrode.

Note that the formation of a vapor layer on the active electrode should lead to the interruption of the current. This makes it possible to analyze the dynamics of the film boiling of the electrolyte on the surface of the electrode. Figure 2B shows an oscillogram of the current flowing through the working electrode near the plasma discharge ignition threshold. As can be seen from the oscillogram, the current interruption time (the drop in the current amplitude from the maximum value to the minimum) occurs in a time of about 20 ms, which corresponds to the propagation velocity (V) of the boiling wave along the active electrode (V ≈ 0.5 m/s). In this case, the maximum value of the average power released in the electrolyte at the moment the current cutoff begins (the threshold of vapor layer nucleation) is approximately 1000 W. These estimates suggest that boiling initially occurs at the end of the electrode and propagates along the electrode at a rate V. This assumption is quite reasonable due to an increase in the current density in the region of the distal end of the active electrode due to edge effects in the distribution of the electrical field.

Let us estimate the maximum increment *∆T* of the electrolyte temperature near the surface of the active electrode, which determines the possible boiling regime on the surface of the active electrode. As can be seen from Fig. 2A,C, the heating time of the electrolyte *∆t* by high-frequency current before entering the current cutoff mode has a value of about *∆t* = 10 ms, which is significantly less than the characteristic time *t* of establishing thermal equilibrium in the electrolyte on scales close in size to the radius of the active electrode: $\tau = \frac{{R^{2} }}{\chi }$, where χ is the coefficient of thermal conductivity of water (*τ ≈ 0.5 s*). This allows us to use a simple relation to estimate the temperature: $\Delta T = \frac{{I^{2} \cdot \zeta \cdot \Delta t}}{{\left( {2 \cdot \pi \cdot R \cdot L} \right)^{2} \cdot c \cdot \rho }}$, where *c* and *ρ* are the heat capacity and density of the electrolyte, respectively, *I* is the magnitude of the effective value of the high-frequency current at the time of its cutoff (*I* = 8A). An estimate of *∆T* gives ≈ 40 °C. Thus, if the current cutoff associated with film vaporization does not begin during *∆t*, then further heating of the electrolyte occurs with the transition to bubble boiling mode on the active electrode.

Furthermore, let us consider under what conditions the transition to the film mode of electrolyte boiling is possible. Let the surface of the water–vapor phase transition move at a speed *V* in the volume of a liquid superheated to a temperature *∆T*_*s*_ along the surface of the active electrode. In this case, the heat flux to the phase transition boundary can be estimated as *q*·*ρ*·*V* = 1.2 × 10^9^ W/m^2^ (q is the specific heat of evaporation of water). Let this flow be provided only due to the flow *c*·*ρ*·*V*·*∆T*_*s*_ from the superheated liquid (*∆T*_*s*_ is the value of superheat). In this case, *∆T*_*s*_ ≈ *q/c*, which corresponds to an unrealistic value of *∆T*_*s*_ ≈ 530 °C. Thus, to ensure the film boiling regime on the surface of the active electrode, as shown in Fig. 2D, an additional heat flux from the vapor phase to the surface of the vapor–liquid phase transition is required. Obviously, in our case, such a flux can be provided only when plasma is burning in the volume of the vapor phase on the surface of the active electrode. In this case, the heat flux from the plasma to the moving front of the phase transition (boiling) should have a value of the order of 1.2 × 10^9^ W/m^2^.

At the moment of the plasma discharge, the temperature of the plasma-forming gas does not exceed 100 °C. Using the relative intensities of the emission lines of atomic hydrogen N_α_ and H_β_ (Balmer series), the temperature of the electron gas in the plasma^25^ at the threshold of its ignition was estimated, which was 0.62 eV. This value of the electron gas temperature indicates that the most effective mechanism of decomposition of water molecules in LTP is the stepwise excitation of vibrational degrees of freedom of water molecules^26,27^. In this case, there is a reaction involving excited H_2_O* water molecules in an act by an unbranched chain mechanism initiated by the dissociation of H_2_O → H + OH. It is essential that the vibrational excitation of water molecules stimulates two-particle chain continuation reactions^26^:

1 $$\begin{aligned} & {\text{H}} + {\text{H}}_{2} {\text{O}}^{*} \to {\text{H}}_{2} + {\text{OH}} \\ & {\text{OH}} + {\text{H}}_{2} {\text{O}}^{*} \to \text{H}_{2} \text{O}_{2} + {\text{H}} \\ & \text{HO}^{\cdot} + \text{HO}^{\cdot} + \text{M}^{\prime} \to \text{M} + \text{H}_{2} \text{O}_{2} \\ \end{aligned}$$

As a result of these reactions, in addition to water vapor, free hydrogen and peroxide vapors appear in the composition of the plasma-forming gas, the partial pressures of which depend on the temperature of the electrolyte near the plasma boundary. Note that, in the case of additional heating of electrons in the anode potential drop zone to 4–5 eV, the energy losses of hot electrons in the water vapor plasma will mainly be determined by inelastic losses due to dissociative adhesion^26,27^. However, such a temperature seems unlikely due to the high density of the plasma-forming gas. As for the temperature of the plasma-forming gas in the stationary regime of plasma burning, it is mainly determined by the heat flux from the active electrode (cathode), which can be heated up to the melting temperature due to ion bombardment by positive plasma ions.

## Results

### LTP radiation spectra when exposed to renal calculi

To confirm the mechanism of the plasma-chemical destruction of renal calculi, a spectral analysis of plasma radiation during its interaction with the surface of the kidney stone was carried out. It is known that the most common kidney stones are calcium stones (calcium oxalate and calcium phosphate), representing 70–80% of the total number of concretions; struvite stones (magnesium and ammonium phosphates), constituting 10–15%; and uric acid stones, constituting 5–15%^1,3^. For this reason, the spectra of characteristic lines of calcium radiation in the radiation of plasma-forming gas when exposed to oxalate stone were studied. In the experiment, a quartz cuvette filled with an electrolyte solution was used. A 0.9% NaCl salt solution (saline solution) was used as the electrolyte. To identify the calcium lines, the plasma spectrum was taken in a solution of CaCl_2_ salt. In experiments on fixing the emission spectra of a plasma-forming gas, a platinum wire with a diameter of 0.5 mm was used as the active electrode, and a carbon plate was used as the anode.

**Figure 3:**
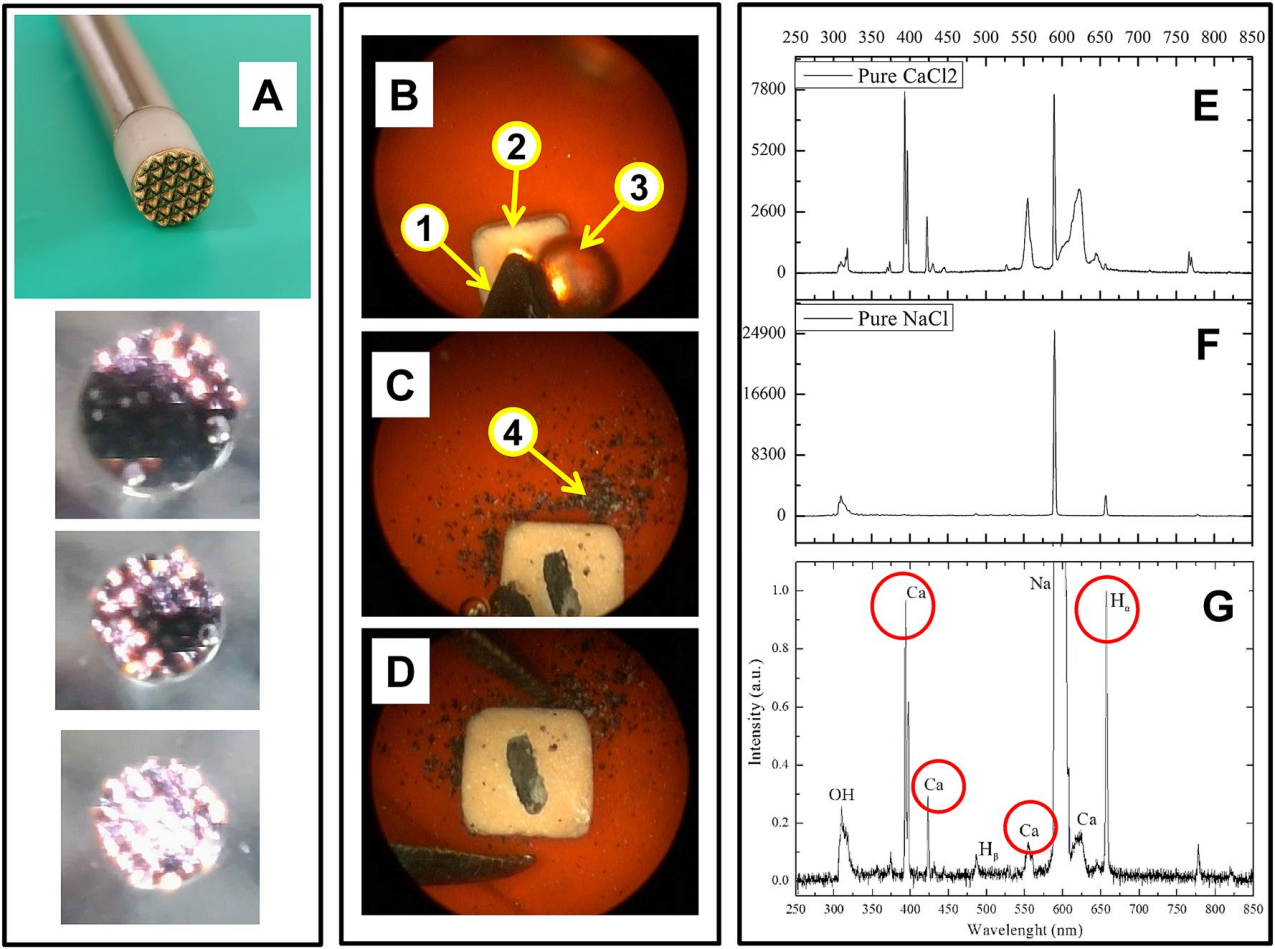
The distal part of the probe for plasmakinetic lithotripsy (**A**): active and passive electrodes. The images of plasma on the active electrode of the probe at the threshold of ignition, intermediate and in the full operating mode are shown (**A**). Operating field views of the stone destruction: initial (**B**), intermediate (**C**), and final stages (**D**). The active electrode (**B1**), a dummy stone (**B2**), and a bubble of hydrogen (**B3**) are shown. The effect of thermic shock wave action on the sample (**C4**). Spectra of pure CaCl_2_ (**E**) and NaCl (**F**), and the spectrum of solution after stone treatment by plasma (**G**).

The objective of the experiment was to identify the spectral lines of atomic calcium, which is one of the main components of renal oxalate stones, and was solved as follows:

1. To isolate the characteristic lines of calcium radiation, the spectrum of plasma radiation in CaCl_2_ solution was recorded. To account for the hydroxyl, sodium and hydrogen lines, a comparison was made with the plasma radiation spectrum^28^.
2. The emission spectrum of the plasma-forming gas was recorded during the plasma exposure to the oxalate stone in order to detect atomic calcium in its composition (Fig. 3).

As a result of plasma exposure to the stone, four intense calcium lines were recorded in the spectrum of the plasma-forming gas, along with the usually fixed lines of hydrogen, sodium and hydroxyl^5–13,16^. An increase in the time of plasma exposure to the stone led to an increase in the intensity of radiation of calcium lines. After removing the stone from the electrolyte solution, calcium lines were also observed in the plasma spectrum. The presence of calcium in the plasma radiation spectra and after exposure to a kidney stone indicates the presence of calcium ions in an electrolyte solution initially free of this element (NaCl). Thus, the results of spectral analysis confirm the mechanism of plasma-chemical decomposition of the renal calculus material and the transition of calcium-containing molecules into the electrolyte.

### Plasmakinetic destruction of renal calculi

**Figure 4:**
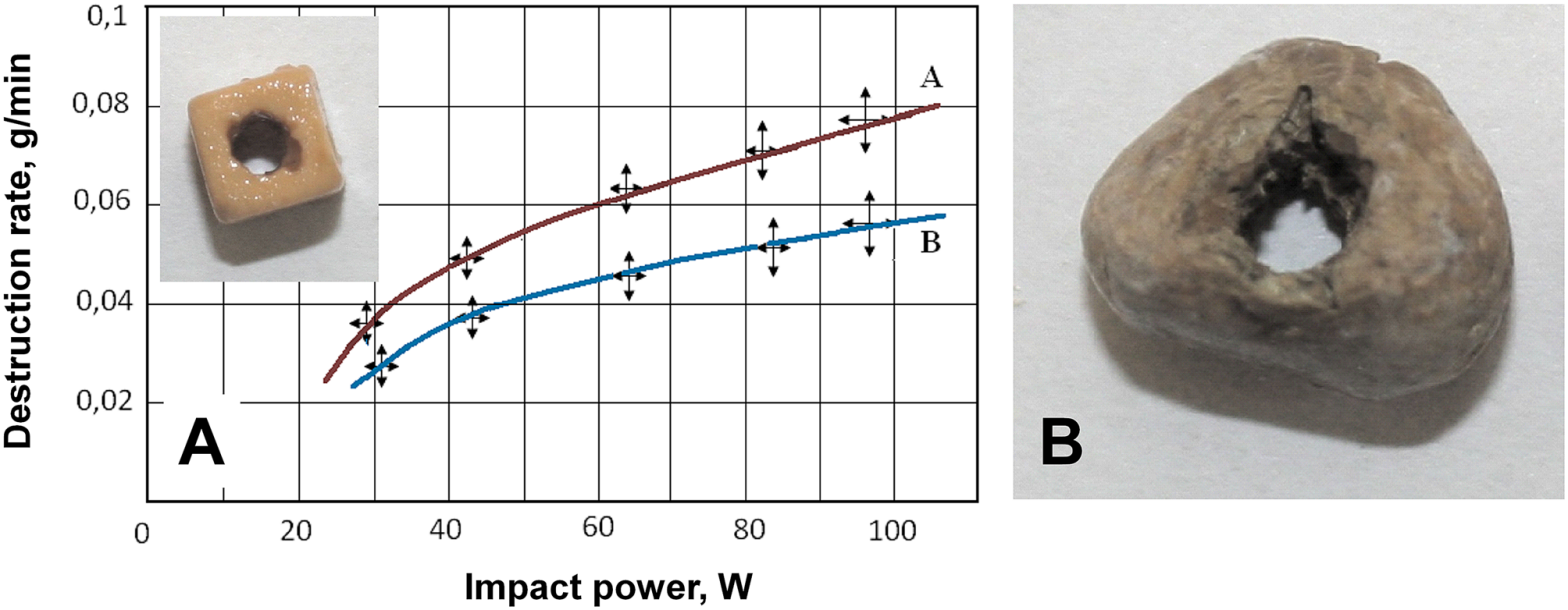
Dependence of the destruction rate (**A**) of oxalate (red) and phosphate (blue) dummy kidney stones on the average impact power. The phase of destruction of the real kidney stone is shown (**B**).

The result of LTP exposure to a calcium oxalate monohydrate type kidney stone (1400–1500 HU) and a dummy kidney stone (1200 HU) after 2 min of exposure is shown in Fig. 4. The depth of the cavity in the stone corresponded to the thickness of the kidney stone and was 4.5 ± 0.5 mm for the dummy and 5.7 ± 0.5 mm for the kidney stone. The rate of deepening of the electrode was approximately 2.5–3.0 mm/min. At the same time, the destruction of stones into particles with a size of no more than 0.5 mm was observed. For example, in the case of a dummy stone, the particle size was estimated by the method of optical microscopy: 136 randomly selected fragments were used to do the size measurements. According to obtained results, particles with a size of 0.35 mm made up the largest part of the sample (46%), followed by particles with a size of 0.4 mm (30%), and then particles with a size of 0.3 mm (13%). The remaining particle sizes are represented by fractions, the contribution of which is less than 7% of the total number.

The effectiveness of plasmakinetic destruction was evaluated in a laboratory experiment on the destruction of oxalate and phosphate stones using a matrix electrode as shown in Fig. 4A,B. The mass loss index ∆M was considered as a criterion for the effectiveness of the impact Mo–M (Mo is the initial mass of the stone, and M is the current mass after exposure to plasma), as well as its velocity dM/dt at different power levels of high-frequency current. The exposure time to assess the rate of destruction was 5.0 min. At the same time, the mode of immersion of the electrode into the stone with transverse movements of the electrode by about half the diameter was carried out. The results of the destruction rate depending on the average power for oxalate and phosphate stones are shown in Fig. 4A.

It follows from the figure that the destruction of the stone depends on the structure and composition of the stone, the power of the impact and, from the point of view of the energy supply, the process of plasmakinetic destruction is a threshold. The presence of a threshold is due to the fact that the LTP excitation has a threshold character, which depends on the size and configuration of the working part of the active electrode.

A feature of unsteady high-frequency discharge LTP is the ability to excite shock waves in the electrolyte solution. This is evidenced by the appearance on the plasma current waveform of short (tt ≈ 1 ns) pulses characteristic of the electrical breakdown of the vapor–gas plasma layer (Fig. 2B,D). The study of the mechanism of generation of these pulses was not part of the objectives of this work. Their appearance is probably due to the hydrodynamic instability of the plasma-electrolyte boundary. As a result of such processes, shock waves may occur in the electrolyte solution medium^29,30^. The electrical breakdown of the vapor–gas layer leads to the formation of cavitation bubbles in the boundary layer of the liquid, which, while expanding, create a shock wave acting on a closely located stone^31^. The repeated shock wave creates compressive and tensile mechanical stresses that lead to mechanical destruction of the stone^32^. However, noticeable shock wave destruction is observed at an increased power of the RF current supplied to the plasma and at the moment of contact of the active electrode with the stone. This leads to the appearance of spark discharges due to the electrical breakdown of the vapor–gas layer. Thus, with an increase in the value of the energy contribution, both processes are responsible for the destruction of the stone, as evidenced by the appearance of calcium-containing molecules in the electrolyte solution, as well as fragments of the destruction of the stone in the form of grains of sand (Fig. 3C,D).

## Discussion

Analysis of the experimental results suggests that plasmakinetic destruction of the stone occurs as a result of several processes, the main of which are plasma chemical and shock wave. At the same time, certain destruction mechanisms prevail depending on the energy parameters of the plasma, the structure of the stone, its chemical composition, and the geometry of the working part of the electrode.

Due to the weak intensity of the high-frequency signal taken from the sensor, measuring the amplitude of a shock wave generated by the plasma presents certain difficulties. The amplitude of this signal is much smaller than the amplitude of electrical pickups generated by plasma. The frequency spectrum of these pickups is in the range from hundreds of MHz to several GHz. Those plasma-generated signals make amplitude measurements from a high-resolution pressure sensor very difficult. Nevertheless, there is indirect evidence confirming that small fragments of dummy renal concretions appeared as a result of mechanical action: the absence of traces of thermal exposure during microscopic examination in the optical range.

### Temperature effects

In laser lithotripsy the temperature of the surrounding fluid can be high if the laser is activated for extended periods. In the case of plasma excitation, the temperature of water solution around the working electrode is very heterogeneous, but not extremely high as in the case of laser. For example, the temperature at the plasma–electrolyte interface does not exceed one hundred degrees due to the presence of a vapor layer on the working electrode. Basically, the intensive mixing of the solution inside the kidney leads to its significant cooling. Direct measurements of the temperature inside the artificial kidney showed that it does not exceed the temperature of the water solution entering the kidney by more than a couple of degrees. If a reversible circulation system is used^33^, the temperature between internal and external solutions becomes practically negligible. It must be stressed that in the experiments performed for this study such a scheme of solution circulation was exactly applied.

### Mechanism of plasma-chemical action

**Figure 5:**
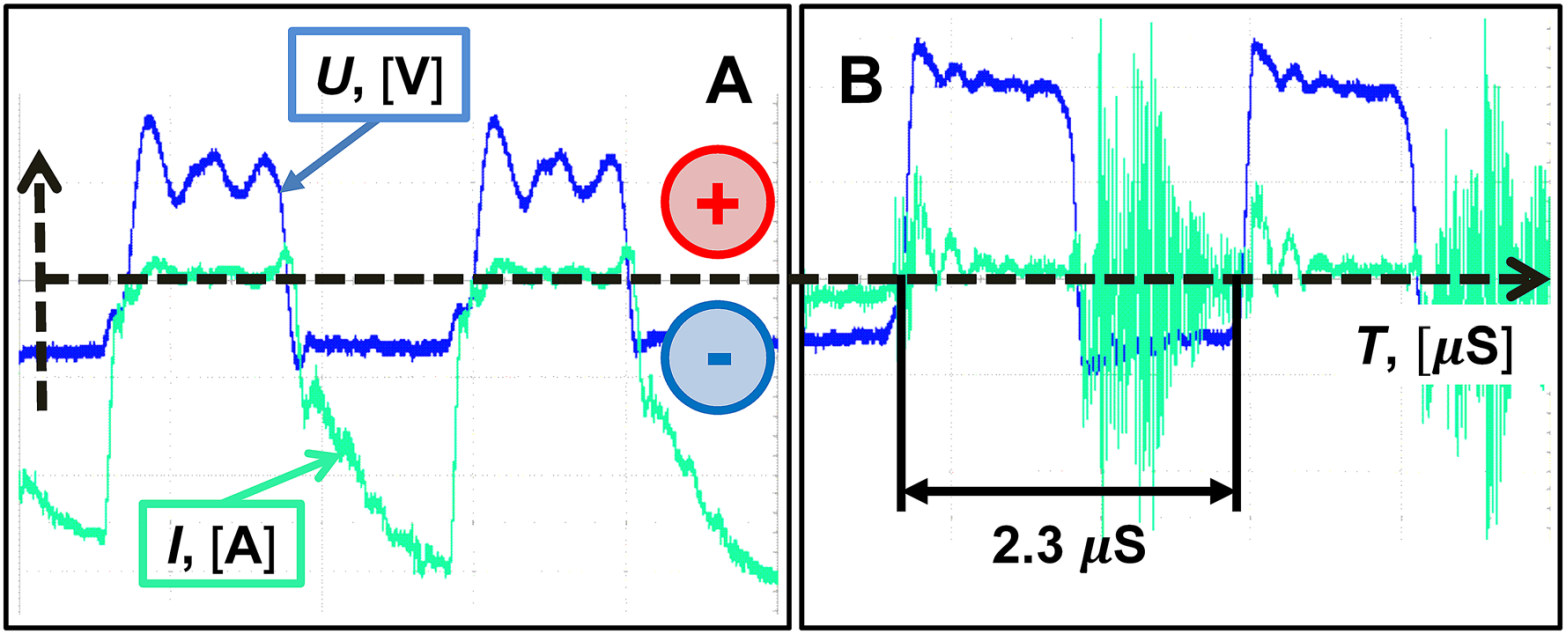
The voltage (blue curve) and current flowing through the plasma (green curve) in operating mode are shown. The steady-state plasma burning mode (**A**); the unsteady plasma burning mode (**B**).

During LTP combustion, the electric field strength in the vapor–gas layer surrounding the metal electrode can reach values of 10^6^–10^7^ V/m^34^. This field strength contributes to the shock emission of electrons from the metal cathode, which is necessary to maintain a steady state of plasma combustion. Typical curves of the voltage between the active and passive electrodes and the current flowing through the plasma in the operating mode are shown in Fig. 5.

The curves (Fig. 5) show that the plasma burns only in the negative half-cycle of the HF current. This indicates the presence of a constant current component, the presence of which makes possible the flow of electrochemical processes in the electrolyte section of the chain.

Let us consider the processes taking place at the plasma-electrolyte interface in a thin boundary layer of the electrolyte. Electrons, as the main charge carriers in plasma, bombard the surface of the electrolyte. Once in the electrolyte, they lose their energy at a depth of no more than a few nanometers, and upon reaching thermal equilibrium with the liquid, they react with hydration to form a hydrated state^34–38^. At the same time, one of the main mechanisms of plasma-chemical action on a solid (kidney stone) can be considered plasma-chemical ablation due to chemical reactions occurring with the participation of hydrated electrons. A method for estimating the concentration of hydrated electrons is given in Appendix A. As a result, considering the dimensional concentration, at the electrolyte-plasma interface (*x* = *R*) for the equilibrium concentration of hydrated electrons, we obtain *N*(*R*) = 3.7 mol/m^3^.

From the expression for *N(x)* (Appendix A of Supplementary Information), it follows that the distance at which the concentration of hydrated electrons drops^39–41^ by an order of magnitude is about 2.5 × 10^−7^ m, which gives 4.0 × 10^−12^ m^3^ for the volume of the active zone. At the same time, the profile of the interfacial concentration reaches a stable distribution in a time of about 4 × 10^−6^ s.

Thus, when the surface of the concrements approaches the plasma-electrolyte phase boundary at a distance of less than 2.5 × 10^−7^ m, plasma-chemical erosion (ablation) of the concrements is possible as a result of interaction with chemically active hydrated electrons.

### Further prospects of the study

As the studies have shown, the plasma-chemical mechanism of action on the constituents of renal calculi is promising in the technology of plasmakinetic lithotripsy. In this case, the plasmakinetic destruction of the stone occurs as a result of several processes, the main of which are plasma-chemical and shock wave. This or that destruction mechanism prevails depending on the energy parameters of the plasma, the structure of the stone, its chemical composition, and on the geometry of the working part of the electrode. In this case, the effect of plasma can produce both plasma-chemical ablation of the stone and its defragmentation into small (sandy) fragments. However, the development of the technology of plasmakinetic lithotripsy requires the solution of a wide range of scientific and technical problems, among which the following can be distinguished: (i) the choice of materials for the active electrode is not only resistant to the effects of plasma in the mode of continuous glow discharge and in the mode of pulsed breakdowns but also to the minimum toxicity of the products formed as a result of this exposure; (ii) selection of the optimal geometry and design of the working part of the active electrode; (iii) selection of power supply modes that ensure stable plasma combustion (glow discharge and pulsed mode); (iv) the distal part of the probe is rigid. Since this study deals only with plasmakinetic destruction of renal calculi, we did not focus on the technical details of related to the improvement of the device design. However, it is obvious to us that the high-frequency leads of a system power supply can be flexible.

Moreover, the choice of parameters and hydrodynamic system for continuous wetting of the plasma exposure zone provide an optimal flow rate of saline solution with a minimum overpressure in the renal cavity. The solution of these questions will make it possible to move on to experimental studies that answer the question of the prospects of the technology of plasmakinetic lithotripsy.

## Conclusions

We found that the dynamical plasma destruction of dummy and real kidney stones was caused by several processes: the plasma induced effect of chemical decomposition of stones and the shock wave effect of electrical breakdowns in the plasma. The average rate of contact destruction of renal calculi can be controlled depending on the plasma generator input power and the time of plasma exposure. Thus, the method of stone fragmentation by high-frequency glow discharge plasma is rather perspective and can be used in endoscopic urology for percutaneous and transurethral lithotripsy. It must be stressed that a potential experimental setup for the high-frequency glow discharge plasma kidney stone destruction will consist only electronic devices, which are obviously cheaper than powerful laser installations, particularly with fibers. Therefore, the implementation of this technique from our point of view will be cheaper than the current laser-based installations.

## Supplementary Information

Supplementary Information.

## Data Availability

All data generated or analysed during this study are included in this published article [and its supplementary information files].
