# Supplementary material for: In vitro renal calculi destruction by a high-frequency glow discharge plasma
Source: Sci Rep. 2022 Jul 25;12:12637. doi: 10.1038/s41598-022-16702-5 (PMC9314433; doi:10.1038/s41598-022-16702-5)
Supplement: Supplementary file 1 — Supplementary Information. [file 41598_2022_16702_MOESM1_ESM.pdf]

Appendix A.

Let us estimate the concentration of hydrated electrons at the plasma-electrolyte interface. A hydrated electron can either undergo a reduction reaction with electrolyte cations or recombine through a diffusion-limited second-order recombination process [34,39,40]:

$$e_{aq}^{-} + e_{aq}^{-} \rightarrow H_2 + 2 \cdot OH^{-} \quad (1)$$

This reaction has a large reaction rate constant (2k ≈ $1.1 \cdot 10^{7}$ m^3/mol*sec), which determines the maximum concentration of hydrated electrons in the reaction zone at the boundary with the plasma, as well as its thickness, which is limited by the diffusion process. The reaction of a hydrated electron with a mother liquor (water) also has a similar efficiency and composition of the reaction products [40]. Note that the reaction of a hydrated electron with electrolyte cations is less effective due to the depletion of cations at the plasma-electrolyte interface. It is the hydrated electrons of the reaction zone that cause highly efficient reduction reactions in the electrolyte in the boundary layer at the plasma-electrolyte interface. For this reason, the inclusion of a solid into the reaction zone can lead to plasmochemical ablation of its surface material due to a heterogeneous chemical reaction. In this case, the main mechanism of such a reaction can be considered the dissociative attachment of an electron to the positively charged center of the molecules that make up a solid (stone). In this regard, an important physical characteristic when considering plasmochemical processes in the boundary layer is the volume W of the reaction zone and the equilibrium concentration of hydrated electrons No in it. To estimate the value of W, we will proceed from the solution of the diffusion equations for hydrated electrons, taking into account the reaction (1). The diffusion processes will be considered in the plane. The transition to a plane can be considered as justified, since all the characteristic features of the obtained solution occur on scales much smaller than the minimum curvature of the plasma-electrolyte interface. Here, we consider the averaged concept of the boundary without considering its possible fast spatial fluctuations due to capillary waves and the development of various instabilities.

$$D \cdot \frac{\partial^2 N}{\partial x^2} - k \cdot N^2 = 0 \quad (2)$$

where N is the concentration of hydrated electrons, D is the diffusion coefficient of hydrated electrons (D = $5 \cdot 10^{-9}$ m^2/sec) [38], k is the constant of the chemical reaction (1), and x is the coordinate measured from the plasma-electrolyte phase boundary, which is normalized to the radius of the active electrode R.

Note that the approach within the framework of equation (2) does not take into account the influence of the gradient electric field associated with the diffusion potential of negatively

charged hydrated electrons and hydroxyl ions. This approximation is justified by the fact that reaction (1) does not change the total number of negatively charged particles, and the mobilities of hydrated electrons and hydroxyl ions are close [35,39]. For this reason, the concentration gradient of negatively charged particles (hydrated electrons + hydroxyl ions) does not undergo sharp changes on the scale of the reaction zone thickness and is determined by the slow drift of OH^{-} ions towards the neutral electrode (anode).

We write the boundary condition for equation (2) as the equality of the flux of ballistic electrons bombarding the electrolyte with the flux of hydrated electrons leaving the electrolyte volume due to diffusion.

$$\frac{I}{e \cdot S \cdot N_a} = \frac{D}{R} \cdot \frac{dN}{dx}, \quad (3)$$

where I is the current flowing through the plasma (I = 2A), S is the area of the active electrode in the plasma combustion zone ($S = 1.6 \cdot 10^{-5}$ m^2), and N_a is Avogadro's number.

Considering the dimensionless concentration $n = G \cdot N$ in (15), where $G = \frac{k \cdot R^2}{6 \cdot D}$ ($G = 2.1 \cdot 10^7$ m^3/mol) for the value N, we obtain the equation:

$$\frac{d^2N}{dx^2} = 6 \cdot N^2 \quad (4)$$

The solution of equation (4) vanishing at infinity has the form: $(x) = \frac{1}{(x+\Delta)^2}$, where Δ is the integration constant determined from the boundary condition (3) $\Delta = 1.1 \cdot 10^{-4}$.

Moreover, the consideration of the diffusion of hydrated electrons in the framework of equation (2) is valid at distances x << 1.

Assuming x = R, we obtain N(R) = 3.7 mol/m^3. The distance at which the concentration of hydrated electrons drops by an order of magnitude is about $2.5 \cdot 10^{-7}$ m. Thus, when the surface of the concretion approaches the plasma-electrolyte phase boundary at a distance of less than $2.5 \cdot 10^{-7}$ m, plasmochemical erosion (ablation) of a kidney stone is possible as a result of interactions with chemically active hydrated electrons.
